# Multi-Path and Group-Loss-Based Network for Speech Emotion Recognition in Multi-Domain Datasets

**DOI:** 10.3390/s21051579

**Published:** 2021-02-24

**Authors:** Kyoung Ju Noh, Chi Yoon Jeong, Jiyoun Lim, Seungeun Chung, Gague Kim, Jeong Mook Lim, Hyuntae Jeong

**Affiliations:** Artificial Intelligence Research Laboratory, Electronics and Telecommunications Research Institute, Daejeon 34129, Korea; iamready@etri.re.kr (C.Y.J.); kusses@etri.re.kr (J.L.); schung@etri.re.kr (S.C.); ggkim@etri.re.kr (G.K.); jmlim21@etri.re.kr (J.M.L.); htjeong@etri.re.kr (H.J.)

**Keywords:** speech emotion recognition, domain adaptation, SER generalization, Korean Emotional Speech Database, ensemble model, multi-path, group-loss, BLSTM network

## Abstract

Speech emotion recognition (SER) is a natural method of recognizing individual emotions in everyday life. To distribute SER models to real-world applications, some key challenges must be overcome, such as the lack of datasets tagged with emotion labels and the weak generalization of the SER model for an unseen target domain. This study proposes a multi-path and group-loss-based network (MPGLN) for SER to support multi-domain adaptation. The proposed model includes a bidirectional long short-term memory-based temporal feature generator and a transferred feature extractor from the pre-trained VGG-like audio classification model (VGGish), and it learns simultaneously based on multiple losses according to the association of emotion labels in the discrete and dimensional models. For the evaluation of the MPGLN SER as applied to multi-cultural domain datasets, the Korean Emotional Speech Database (KESD), including KESDy18 and KESDy19, is constructed, and the English-speaking Interactive Emotional Dyadic Motion Capture database (IEMOCAP) is used. The evaluation of multi-domain adaptation and domain generalization showed 3.7% and 3.5% improvements, respectively, of the F1 score when comparing the performance of MPGLN SER with a baseline SER model that uses a temporal feature generator. We show that the MPGLN SER efficiently supports multi-domain adaptation and reinforces model generalization.

## 1. Introduction

Human speech is a natural communication method in human–computer interaction (HCI) and human–robot interaction (HRI). Speech emotion recognition (SER), which is based on natural human language, is a key method used to recognize individual emotions in everyday speech. SER uses the acoustic features of a speech segment, not the lexical features having the semantic information of the segment [1]. Hence, it recognizes subjects’ emotions from “how” they speak rather than the content of their words. The predicted emotional context of a target speaker can then be used as an important factor for decision making in intelligent HCI and HRI services [2,3].

Prior to deploying SER models in real applications, the lack of SER databases tagged with emotion labels must be addressed, because they are not sufficient for training deep-SER models. Another challenge is the limited generality of the SER model, owing to the high variability of the acoustic signals of the emotional speech samples.

Emotions have characteristics of high subjectivity and diversity, depending on the individual or culture. Therefore, it is time-consuming and expensive to build a large-scale emotional database annotated with reliable gold-standard emotion labels via human observation. Most SER datasets having gold-standard labels contain thousands of speech samples collected from a limited number of speakers in a specific environment [4,5,6,7]. Therefore, the performance of an SER model trained on single-domain samples is inherently degraded when applied to unseen domain samples that reflect different languages, cultures, speakers, genders, microphone types, positions, and signal-to-noise ratios [8,9,10]. This study defines a single SER domain dataset collected using one collection procedure at one place using the same collection device.

Many studies have effectively utilized limited emotion databases to improve the SER performance. In addition to the typical augmentation methods of speech samples [11,12], there exists a domain adaptation method that utilizes speech datasets already established in the unknown target domain [8,9,10,13,14,15,16]. In comparison with the results of data augmentation in a single domain, it is difficult to guarantee good performance because of the high variability of the acoustic features of the emotional speech samples in the domain [8,9,10,13,14]. However, domain adaptation based on multi-domain datasets can be used to construct better SER models to support such generalities without overfitting.

We propose a multi-path and group-loss-based network (MPGLN) for SER, which supports supervised domain adaptation in multi-domain datasets acquired from multiple environments. The proposed MPGLN for SER (MPGLN SER) is based on an ensemble learning structure for multi-level embedding vector learning for speech segments. It includes a temporal embedding feature generator, transferred feature extractor, and prediction function network that classifies the emotion labels based on the generated and extracted feature vectors. The bidirectional long short-term memory (BLSTM)-based temporal feature generator network learns an embedding vector as a 74-D input of handcrafted low-level descriptions (LLD) of a speech segment. The transferred feature extractor creates feature vectors from the pre-trained VGG-like audio classification model (VGGish) [17], and the proposed MPGLN SER is trained based on multiple losses by the association between the discrete and continuous dimensional emotion labels [1] of the multi-domain samples.

The proposed MPGLN SER is evaluated over five multi-domain SER datasets: the benchmark English Interactive Emotional Dyadic Motion Capture (IEMOCAP) dataset [7], which was widely used in previous studies for SER model evaluation, and the four Korean Emotional Speech Database (KESD) datasets that are built for this study. 

In our evaluation, we use an SER model comprising a BLSTM-based temporal feature generator and the MPGLN predicting network, excluding transferred features, as our baseline model. We then verify the reliability of the baseline SER model using the IEMOCAP dataset. Comparing it with the performance of the baseline SER model, it is confirmed that the proposed MPGLN SER is effective in supporting supervised multi-domain adaptations and reinforcing generalizations [18] of the SER model in multi-domain datasets.

This paper is organized as follows. In Section 2, we present a brief overview of related SER and domain adaptation works. Section 3 describes the proposed MPGLN, which supports multi-domain adaption of SER in multi-domain datasets. Section 4 details the evaluation results of the MPGLN SER, and Section 5 concludes this study and suggests future works.

## 2. Related Works

Recent SER models based on deep-learning architectures [19,20,21,22,23,24,25,26,27,28,29,30] have demonstrated state-of-the-art performance with an attention mechanism [19,20,22,23,25,26]. The deep-learning architectures adopted in previous studies included recurrent neural networks (RNN) [19], convolutional neural networks (CNN) [24], and convolutional RNNs (CRNN) [20,26]. Liu et al. [21] presented an SER model of a decision tree for an extreme learning machine having a single hidden-layer feed-forward neural network, using a mixture of deep learning and typical classification techniques. 

The input features for deep-learning-based SER models are generally extracted from the time or spectrum axis in units of speech segments or frames. There are various LLDs and high-level statistical functions of the LLD single features [19,20,31,32,33]. The spectrum LLD features of speech signals include logMel filter-banks and mel-frequency cepstral coefficients (MFCC). Zero-crossing rates and signal energies are representative time-domain features [27,28,29,30], whereas spectral roll-off and spectral centroid are classified as spectral parameters [33]. A set of multiple single features for acoustic signal processing, such as the extended Geneva Minimalistic Acoustic Parameter Set [34] and the INTERSPEECH 2010 Paralinguistic Challenge (IS10) dataset [35], is now accessible from open-source frameworks, such as OpenSmile [36]. Some studies have investigated the mechanism of modeling and integrating of temporal acoustic features to improve the performance of speech emotion recognition or audio classification [31,32]. Jing et al. [37] presented an evaluation of multiple acoustic feature sets that combined features generated from the pre-trained acoustic model [15,17,38,39].

A typical deep-learning model requires large-scale samples for training. Unfortunately, SER datasets annotated with emotion labels are scarce. Furthermore, collecting SER speech samples and tagging them with emotion labels is time-consuming and expensive. Thus, to overcome the limitations of volume and diversity of labeled speech samples for deep-learning SER models, studies have been performed using data augmentation [11,12,40,41,42], active learning [12,43] based on collected datasets, and domain adaptation [8,9,10,13,14,15,16] to adapt the existing SER datasets to the target domains.

Park et al. [11] presented a data augmentation experiment for speech samples using warping and masking in a frequency channel with a time step. Chatziagapi et al. [40] proposed a method that used generative adversarial networks [44] to extract artificial spectrograms of augmented data to balance each emotion class. 

Active-learning methods have been used to present greedy selection methods of speech samples to construct an initial SER model suitable for a target speaker based on limited samples [12,43]. Abdelwahab et al. [43] proposed the active learning of greedy sampling to select the most informative samples to improve the performance of DNN-based SER models. In a study by Bang et al. [12], samples that were close to the target speaker’s samples in the embedding space were selected; the synthetic minority oversampling technique was applied to increase the number of samples of the minority class.

Domain adaptation techniques are actively being studied in the field of visual classification [18,45]. Metric-based learning is a representative method of learning distances containing the features of inter-domain and -class samples to minimize domain mismatches between the source and target domains. Gao et al. [46] proposed an acoustic model based on ResNet [47] for acoustic scene classification; its learning process is such that it is difficult to distinguish the domain to which a sample belongs.

The domain adaptation for SER models based on multi-domain datasets has the purpose of building an SER model that is not overfitted to a specific dataset and is generalized for unknown target-domain speech data. However, the SER model based on multi-domain datasets has a different applicability from the case that applies data augmentation by oversampling a single domain dataset. It does not guarantee the SER performance improvement, even if several multi-domain speech samples are used to train the SER model, because there is high domain discrepancy in the speech signal, which depends on the collection environments [8,9,10,13,14]. 

Liang et al. [9] proposed a structure that learned emotion-salient features based on audio and video data through an adversarial learning framework, generating embedding features for the purpose of reducing domain discrepancies. Huang et al. [13] presented a network model that aligned the distribution shift in the intermediate feature space between the source and target domains. Neumann et al. [14] introduced an adaptive technique to fine-tune the weights of SER neural networks trained in the source domain using a small number of samples from the target. By using the transferred features from the pre-trained model, Li et al. [15] demonstrated improvements in the SER performance using additional embedding vectors extracted from the pretrained VGGish in AudioSet [48]. Lee et al. [16] presented the generalization effect of emotion recognition by applying dropout and normalization methods in multilingual heterogeneous datasets.

## 3. Ensemble Learning Model for SER in Multi-Domain Datasets

**Figure 1:**
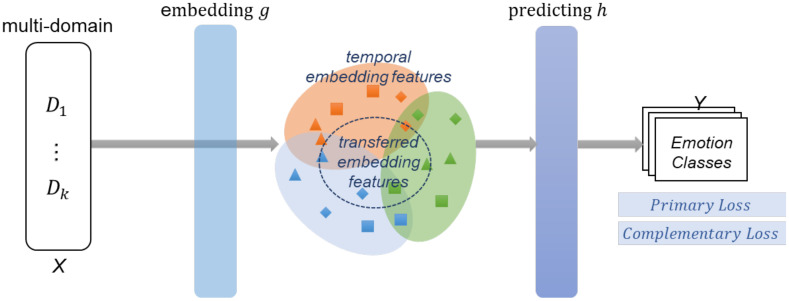
Supervised multi-domain adaptation of the multi-path and group-loss-based network (MPGLN) speech emotion recognition (SER). The model generates the temporal embedding feature and the transferred embedding feature for the speech segment and learns based on multiple losses.

We propose an ensemble learning model to improve the performance of SER generalization in multi-domain datasets. The operational flow of the supervised multi-domain adaptation of the proposed MPGLN SER is shown in Figure 1. We denote speech-input samples and class-label spaces as X and Y, respectively, and the domain datasets are $D=\{D_{1},\;D_{2},\;\ldots ,\;D_{k}$}. This study assumes a supervised learning environment wherein each domain sample has common emotion labels. In this study, each domain dataset consists of pairs $D_{k}={\left\{\left(X_{i}^{k},\;(y_{i\_d}^{k},{\;y}_{i\_v}^{k})\right)\right\}}_{i=1}^{N_{k}}$, where $N_{k}$ is the number of speech samples of the *k*-th domain dataset, and datasets in each speech sample have multiple Y labels. The discrete emotion label is $y_{i\_d}^{k}$ (e.g., “happy” and “sad”), and that of the valence-level is $y_{i\_v}^{k}$ in the continuous dimensional emotion model.

The source-domain dataset used for model training is domain $D_{s}$, and the domain to which test samples to be predicted belong is the target domain, $D_{t}$. There are variant shifts and domain discrepancies of the feature distribution, $d\left(X^{S}\right)$ and $d\left(X^{T}\right)$, of data samples of different domain datasets, $D_{s}$ and $D_{t}$, respectively [45]. 

The goal of the SER model is to learn the classifier function, f:X→Y, in the target domain. Function f consists of the composition of two functions, f=h ° g, where g is an embedding feature generator from the input data space, X, to an embedding feature space, and h is the function used to predict the embedding feature to label-space Y.

**Figure 2:**
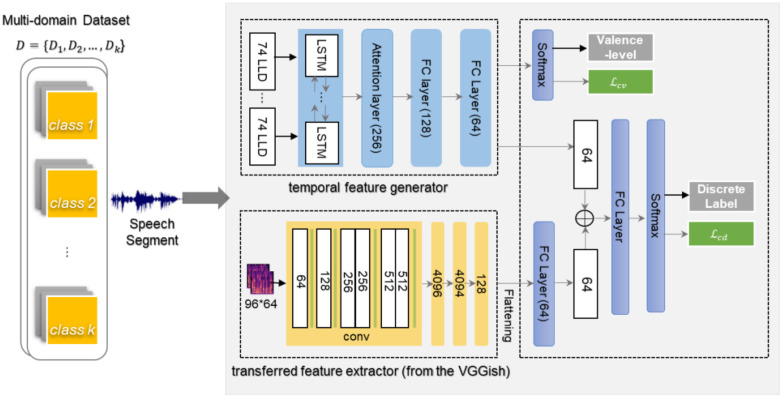
Architecture of the multi-path and group-loss-based network for SER. The MPGLN SER model comprises a bidirectional long short-term memory (BLSTM)-based temporal embedding generator and a transferred feature extractor from the VGG-like audio classification model (VGGish) and its prediction function.

Figure 2 shows the architecture of the proposed MPGLN SER, which generates the multi-level embedding vectors from the multi-path generators. The BLSTM-based feature generator, $g_{BLSTM}$, generates a temporal embedding vector, and the transferred feature extractor, $g_{vgg}$, extracts a transferred embedding vector from the pre-trained VGGish model [17]. 

In the prediction function, h, of the proposed ensemble structure, discrete emotional labels are classified based on the fusion of multi-path embedding vectors from $g_{BLSTM}$ and $g_{vgg}$. It also includes a dimensional valence-level classification function based on the temporal embedding feature generated by $g_{BLSTM}$.

### 3.1. Multi-Path Embedding Features

In this study, the speech segments of an utterance unit are embedded in the feature space through $g_{BLSTM}$, a temporal feature generator of the ensemble structure, and $g_{vgg}$, a transmitted feature extractor. In Figure 2, the temporal feature generator, $g_{BLSTM}$, of the BLSTM architecture reflects a characteristic of the temporal relevance of before-and-after speech features. The 74-D LLD-per-frame speech segment comprises a 13-D MFCC and 40-D Mel-spectrogram, along with 21-D time- and frequency-domain LLDs such as zero-crossing rate, energy, spectral centroid, and spectral roll-off. The 74-D LLD are extracted by the frame that applies sliding windows of 200 ms with a 50% shift in the speech segment. Each speech segment is padded with a zero value to have a fixed number of 100 frames, and the sequence of 100 × 74 per segment is input to $g_{BLSTM}$. The padded input sequence is fed into the $g_{BLSTM}$, comprising 128 cells in each direction, and $g_{BLSTM}$ produces a 256-D feature vector. 

The feature generator, $g_{BLSTM}$, adopts an attention mechanism and focuses on those more discriminative parts of the BLSTM output sequence before activation of the final emotion classification. The attention mechanism for SER assumes that there are certain words and salient parts that express emotions well in the speech segment. Using the attention method, it gives more weight to relevant speech frames of an utterance-level segment for emotion recognition. 

The attention layer focuses on relevant parts of the output sequence of the BLSTM by giving different weight scores and generates the high-level features (hf). It computes weight $\alpha_{t}$ using the softmax function via the attention layer (see Equation (1)), where the BLSTM output vector is $h_{t}=[\vec{h_{t}},\;\overset{\leftarrow }{h_{t}}]$ at time t. It produces the high-level feature, hf, which is the weighted sum, $h_{t}$, obtained by multiplying the weights, $\alpha_{t}$ (see Equation (2)). The generated hf is transited again to an embedding feature vector of $R^{64}$ through the two fully-connected (FC) layers in the MPGLN.

(1) $$\alpha_{t}=\frac{\exp\left(W\cdot h_{t}\right)}{\sum_{t=1}^{T}\exp\left(W\cdot h_{t}\right)}$$

(2) $$hf=\sum_{t=1}^{T}\alpha_{t}\cdot h_{t},$$

The temporal feature generator, $g_{BLSTM}\;:\;X\to R^{64}$, generates a 64-D embedding vector from the input of the 74-D LLD in units of speech-segment frames. The feature generator, $g_{BLSTM}$, in the MPGLN SER can operate as an SER model alone by combining the prediction function, $h_{d}^{baseline}:R^{64}\to Y(y_{i\_d}^{k}$), without using the transferred features from the VGGish. This study uses the BLSTM-based SER model as a baseline for the evaluation of the MPGLN SER. 

The transferred feature extractor, $g_{vgg}\;:\;X\to R^{VGGish}$, extracts the transferred feature vector of data-sample X using the VGGish model. The input speech segment is divided into non-overlapping 960 ms time-unit frames, and 64 mel-spaced spectrogram features that apply a 25 ms window every 10 ms in each frame are extracted using the VGGish model [17]. Using the transferred feature extractor, $g_{vgg}$, it generates a 128-D embedding feature vector from the VGGish model for the speech segment by inputting a frame-by-frame spectrogram in units of 96 × 64. The extracted 128-D embedding vector passes through the fattening and FC layers and is transited to a 64-D embedding vector.

### 3.2. Group Loss

Equation (3) shows how classifier f is trained on the classification loss, $L_{c}\left(f\right)$, of the emotion labels Y of the speech samples X, where ℓ is an appropriate loss function similar to cross-entropy for multi-class classification [45,49].

(3) $$L_{c}\left(f\right)=\ell \left(f\left(X\right),Y\right)$$

The proposed MPGLN SER is trained to simultaneously minimize multiple losses, which are induced by the association of multi-dimensional emotion labels. The discrete emotion labels are intuitive for expressing the emotion, but it has difficulty in expressing complex emotions. The dimensional emotion labels are capable of normalized expressions of complex emotions. However, doing so, it is difficult to intuitively distinguish emotions at similar positions (e.g., “fear” and “anger”) in the arousal-valence axis [1]. This study derives an association between discrete and dimensional valence-level labels based on real SER domain datasets and applies a method of simultaneously learning the loss for each emotion-label classification in the MPGLN model.

As shown in Figure 2, the MPGLN SER learns simultaneously based on the two losses: $L_{cv}$ for the valence-level label using the $R^{64}$ feature vector generated from $g_{BLSTM}$ and $L_{cd}$ for predicting the discrete emotion label. 

The primary loss, $L_{cd}$, is used for the predicting function, $f_{d}=h_{d}\;{}^{\circ}\;\left(g_{BLSTM}⊕g_{VGGish}\right)$, where $h_{d}\;:\left(R^{64}⊕R^{VGGish}\right)\to Y(y_{i\_d}^{k}$) predicts the discrete emotion label of $y_{i\_d}^{k}$ via the combination of two embedding vectors. The complementary loss, $L_{cv}$, is that of the predicting function, $f_{v}=h_{v}\;{}^{\circ}g_{BLSTM}$, which classifies the valence-level labels, where $h_{V}\;:R^{64}\to \;Y(y_{i\_v}^{k}$). Equation (4) shows that the proposed MPGLN SER is trained to minimize group loss $L_{g}$ about the prediction functions, $f_{d}$ and $f_{v}$:

(4) $$L_{g}=\mathrm{Group}(L_{cd}\left(f_{d}\right),\;L_{cv}\left(f_{v}\right)).$$

## 4. Evaluation

### 4.1. Datasets

We evaluated the proposed model using five multi-domain datasets contained in three real SER databases. For the evaluation of the MPGLN SER based on multi-cultural datasets, two KESD databases (i.e., KESDy18 and KESDy19) constructed for this study, and the IEMOCAP are used. KESDy18 and KESDy19 comprise two domain datasets based on heterogeneous microphone devices. 

In the IEMOCAP dataset, data were collected from the scenarios for inducing the five target emotions (“happy”, “sad”, “neutral”, “angry”, and “frustration”), and annotators selected one of the six basic emotions (“angry”, “sad”, “happy”, “disgust”, “fear”, and “surprise”) [50] along with “frustration”, “excited”, and “neutral” as the discrete emotion labels. Numerous data were annotated with the emotion categories such as “fear” and “disgust”, which do not belong to the target emotions in IEMOCAP [7]. Even in the KESD database, considering the subjectivity and diversity of human emotion perception, the categorical emotion label was tagged as one of the six basic emotion labels along with “neutral”.

**Figure 3:**
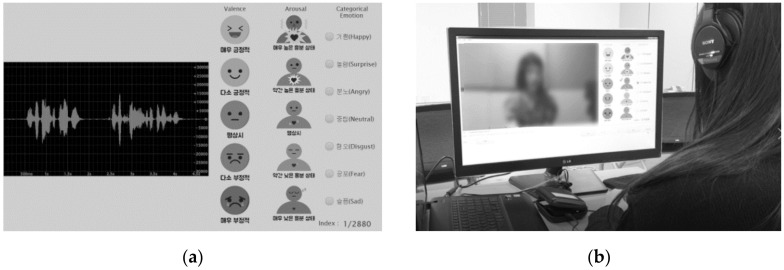
External annotator tags the emotion labels for speech segments using the tagging application while watching the recorded video and listening to the Emotional Speech Database (KESD) speech segments: (**a**) evaluating emotional labels of KESDy18 via the tagging application; (**b**) evaluation of the KESDy19 speech segments looking at the recorded video.

The KESDy18 comprises speech samples in which 30 voice actors uttered 20 sentences while expressing the four given emotions of “angry”, “happy”, “neutral”, and “sad”. The six external taggers evaluated the speech segments while listening to the recorded utterances as shown in Figure 3a. The annotators tagged one of the seven categorical emotion labels comprising the six basic emotions [50] in addition to “neutral”, whose tagged labels are more diverse than the classification of the actor’s expressed emotion. They tagged labels of arousal and valence-level on a five-point scale for each segment. The final categorical emotion label was determined by majority vote. The label of arousal and the valence-level were determined from the average value of the levels tagged by the evaluators. KESDy18 simultaneously collected speech data from two heterogeneous microphones (i.e., a cell-phone’s built-in microphone (PM) and an external microphone (EM) connected to a computer). According to the type of microphone devices, KESDy18 comprised the KESDy18_PM dataset plus the KESDy18_EM dataset.

The KESDy19 includes the speech samples of 40 voice-actors who speak Korean as their native language using collection scenarios similar to those of the IEMOCAP. KESDy19 consists of 20 sessions collected from speech and electrocardiogram signals produced during the dyadic acting of two voice actors, the process of acting was recorded. Each session consists of 10 plays having lengths of 4–10 min. Six plays were based on scenarios written to induce specific emotions, and the other four were improvised during the dyadic interactions. Each speech segment per speaker was tagged using one of seven categorical emotion labels, and the average value of the five-point scale of arousal and valence-level was annotated by 10 external taggers using the same tagging application as shown in Figure 3b. KESDy19 comprises a KESDy19_EM dataset that used an external microphone and a KESDy19_PM dataset that simulated the KESDy19_EM dataset via a cell-phone’s microphone.

The IEMOCAP is a widely used SER performance evaluation model organized into five sessions of multi-modal audio, visual, and textual data taken from interactive dyadic interactions performed by 10 voice actors. In each session, two voice actors emotionally performed improvisations or scripted scenarios. The speech segments of their utterance-levels were tailored to discrete emotion labels of “happy,” “sad,” “neutral,” “angry,” “surprise,” “frustration,” “excited,” “disgust,” or “fear” based on the majority opinions of three external human annotators. The IEMOCAP data were also tagged with labels of arousal and valence based on a five-point dimensional emotion scale [39,51]. The IEMOCAP database provides the re-rounded average score of the evaluations of arousal and valence-levels according to the five-point scale based on evaluations by six external evaluators. Many prior studies evaluated SER performance using the IEMOCAP database to classify the four emotion categories of “happy,” “sad,” “neutral,” and “angry.” 

**Figure 4:**
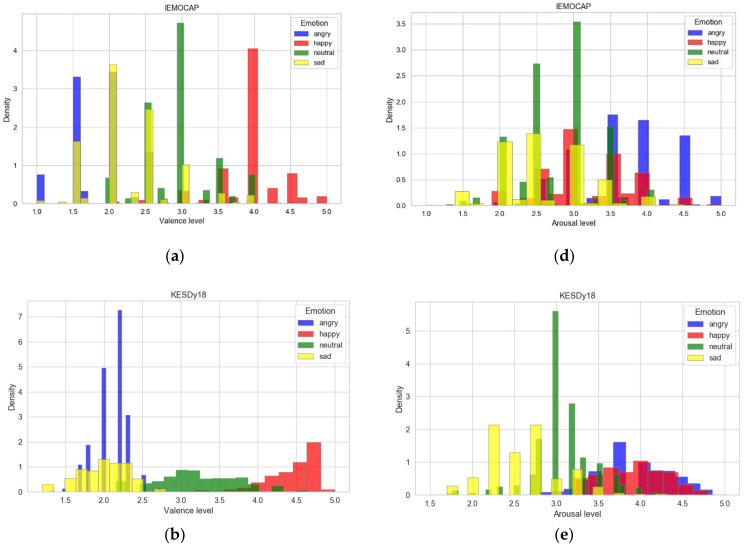

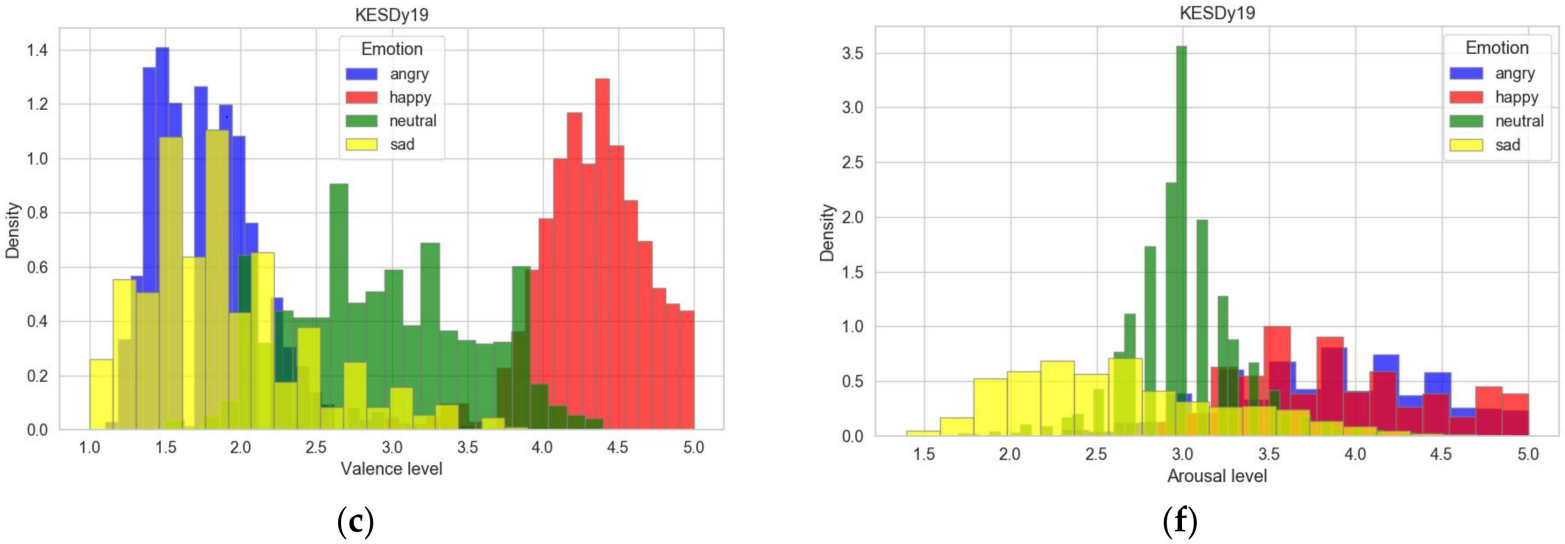
Distribution between discrete and dimensional emotion labels of the five-point scale: (**a**) distribution of discrete and valence-level labels of Interactive Emotional Dyadic Motion Capture database (IEMOCAP); (**b**) distribution of discrete and valence-level of KESDy18; (**c**) distribution of discrete and valence-level of KESDy19; (**d**) distribution of discrete and arousal-level of IEMOCAP; (**e**) distribution of discrete and arousal-level of KESDy18; and (**f**) distribution of discrete and arousal-level of KESDy19.

Figure 4 shows the distribution of four discrete emotion and arousal/valence-level labels on the five-point scales of IEMOCAP, KESDy18, and KESDy19. As shown in Figure 4a–c, the speech samples of the “happy” class are distributed at the highest valence level, and the “neutral” samples are in the middle. The speech data labeled with “sad” and “angry” classes show a distribution of low-level valences across all three SER databases. The association between discrete emotion labels and those of arousal-level shows more irregularities in Figure 4d–f. The speech samples tagged with the “sad” class are distributed in the overall arousal-level, and the samples of the IEMOCAP with the “happy” label are distributed in the overall level of arousal, unlike the other two KESD.

In Figure 4, the speech samples corresponding to the discrete emotion classes constitute roughly three distribution groups across the label of valence-level. The three distribution groups are “happy,” “neutral,” and “sad” or “angry.”

In this study, we mapped the valence-level labels of the five-point scale to a three-point scale using the induced association between discrete and dimensional emotion labels, as shown in Table 1 and Figure 4. Each valence-level (i.e., 1, 2, and 3) of the three-point scale represents “negative”, “neutral”, and “positive” emotional states, respectively. For the conversion to the valence-level of the three-point scale, this study assigned sample labels of valences less than 2.5 to the first valence-level, samples of 4.0 or higher to the third, and the others to the second, respectively. Table 1a shows the mean and standard variation of arousal and valence-levels on a five-point scale for each discrete emotion category. Table 1b shows the confidences of association [52] of the speech samples of four discrete emotion classes included in the valence levels of the three-point scale. The confidence $Conf.\left(C_{i}\to V_{j}\right)=\frac{N_{C_{i}\cup V_{j}}}{N_{C_{i}}}$, where ${\;C}_{i}$ is the discrete emotion label, 1≤i≤4, and $V_{j}$ denotes the valence-level, 1≤j≤3.

Table 2 shows properties of the five domain datasets of three SER databases used for the evaluation, where we used speech segments having lengths of 2 s or longer as one of four categories of emotion labels, “angry”, “happy”, “neutral”, and “sad.”

### 4.2. Evaluation of the BLSTM-Based Baseline SER

As shown in Table 2, the five domain SER datasets used for evaluation were unbalanced in the number of samples of the discrete emotion classes. We did not apply oversampling, data augmentation [11], or weighted loss methods [46] to minority classes for objective verification of the proposed MPGLN SER. 

Speech samples of each class in the multi-domain datasets were trained in the SER model by the units of the speech segment, which consisted of the voiced part of the vocal-cord vibrations and unvoiced parts such as a silence section between voiced parts [53]. This study did not remove the unvoiced region from any speech segment. However, it framed the entire voiced and unvoiced parts of the segment as input to the model.

We present four performance metrics in consideration of the sample imbalance of each emotion class: weighted accuracy (WA), unweighted accuracy (UA), precision (PR), and F1 score. WA is the overall accuracy, calculated as the ratio of the total number of test data and the number of samples accurately predicted by the actual label. UA is calculated as the average of the recall values of four classes and is an important performance indicator in the evaluation of the SER model based on imbalanced datasets [19,20,26].

This study applied *z*-normalization [1] of the means and standard deviations of each dataset to reduce the fluctuations of the speaker and speech signals. We evaluated the speaker-independent leave-*p*-subjects-out (L*p*SO) validation technique, where p is the number of subjects to leave out when training the model. For training, we used separated samples belonging to speakers accounting for 80% of the total number in each dataset; samples of the remaining 20% were evaluated as test data. 

For the evaluation of IEMOCAP, we used a leave-two-subjects-out evaluation that applied speech data from two speakers participating in one session as the test data, which was the leave-one-session-out (LOSO) validation. KESDy18 was evaluated as a leave-six-subjects-out sample from the set of 30 speakers. The evaluation of KESDy19 was conducted as a leave-eight-subjects-out sample for four sessions of the 20 sessions played in pairs by 40 speakers. The training and test data separated for speaker-independent evaluation in each dataset were equally applied to the evaluation of a single domain, multi-domain, or domain generalization, as shown in Table 3 and Table 4 and Tables 6–8.

In the evaluation of this study, a model based on the temporal embedding features and the learning loss, $L_{cd}$, without the transferred embedding feature was assumed to be the baseline SER model. It can be seen that this baseline operated using a single-path-single-loss (SPSL) scheme. In the evaluation, the proposed MPGLN and the baseline SPSL SER model were trained with a batch size of 200 samples at 25 epochs using an Adam optimizer and a drop rate of 0.6 to the last two FC layers. The learning rate of the optimizer was $1.10^{-3}$. The model was evaluated over 10 iterations of training and testing, and the final value of each performance metric was calculated as the average value.

The baseline SPSL SER model uses the 74-D LLD integration per-frame of speech segment, which comprises 13-D MFCC and 40-D Mel-spectrogram (Mel-spec), along with 21-D time- and spectral-domain (TimeSpectral) LLDs such as zero-crossing rate, energy, spectral centroid, and spectral roll-off. We evaluated the performance of each combination of LLDs with our baseline SER model based on multiple SER datasets. Table 3 summarizes the performance evaluation according to the input feature set of the LLDs used in this study, as shown in the evaluation results based on the IEMOCAP, KESDy18_EM, and KESDy19_EM datasets. It can be observed that MFCC is the dominant feature of SER from the results in Table 3. The SER performance improved from 1.6% to 3.2% based on the F1 score in comparison with the single input of MFCC when using the input combination of MFCC and Mel-spectrogram, along with TimeSpectral LLDs.

Table 4 shows the results of the speaker-independent evaluation of the BLSTM baseline SPSL when classifying the four discrete emotion labels in each of the five domain datasets. The evaluation based on KESDy19 showed similar performance results as IEMOCAP. In the evaluation of KESDy18, it showed higher performance results than the other two databases. 

A previous study by Zheng et al. [54] demonstrated the performance of 40% WA of the CNN-based SER model for the five emotion classes based on IEMOCAP. For a fair comparison of the SER performance, this study performed a comparison with the previous RNN-based SER models that presented the UA performance of the four emotion classes based on IEMOCAP, which was the test environment in many previous SER studies. 

In Table 5, we compare the performance results of previous RNN-based SER models and the SPSL baseline model in the LOSO evaluation to classify the four emotion labels based on the IEMOCAP dataset. These studies present a UA metric of the average recall for each emotion class, considering the imbalance of the number of samples. As shown in Table 5, our baseline BLSTM SER model achieved a competitive performance of UA 59% in the LOSO validation based on IEMOCAP.

### 4.3. Evaluation of Multi-Domain Adaptation

As shown in Table 6, Table 7 and Table 8, evaluations were performed using a single-domain evaluation, a multi-domain adaptation, and a multi-domain generalization according to the source and target domains participating in training and evaluation. The division of training and testing data separated for speaker-independent evaluation in each dataset used the same configurations as those used in Table 3, Table 4, Table 5, Table 6, Table 7 and Table 8. In Table 6, Table 7 and Table 8, the highest F1 scores are highlighted.

Table 6 shows the evaluation results when classifying four discrete emotion classes based on each of the five domain datasets. The evaluation was conducted in three experimental environments according to the type of SER model: The baseline SPSL model learns from the temporal embedding features and the single-loss $L_{cd}$. Multi-path-single-loss (MPSL) uses multi-path embedding vectors and is trained only on $L_{cd}$ without the complementary loss, $L_{cv}$, for valence-level classification. Multi-path-group-loss (MPGL) learns from multi-path embedding vectors and the group loss, $L_{g}$, consisting of $L_{cd}$ and $L_{cv}$. 

When compared with the harmonic-mean F1 score based on the KESDy18_PM dataset shown in Table 6b, the performance of the SER of the MPSL using a single-loss $L_{cd}$ showed an improvement of 1% over that of the baseline SPSL. The SER MPGL model trained on the loss group, $L_{g}$, showed an F1 improvement of up to 3.7% over the SPSL’s F1.

Table 7 shows the results of multi-domain adaptation evaluation when the SER model was trained with samples aggregated from multiple-domain SER datasets collected from various environments. The separated test samples for about 20% of the speakers were evaluated for speaker-independent evaluation. As shown in Table 7a, regarding KESDy18, which consisted of two datasets collected simultaneously via heterogeneous devices, the proposed SER model trained on the group-loss $L_{g}$ of MPGL achieved an F1 improvement of up to 3.7% over the baseline SPSL.

Table 8 presents the evaluation results of the proposed MPGLN SER for supporting multi-domain generalization. In the evaluation of Table 8a, the SER model was trained with the aggregated samples of KESDy18_PM, KESDy18_EM, and KESDy19_EM datasets and was evaluated against the separated test samples of the KESDy19_PM domain, which was not used for training but was collected from the same language culture. The evaluation results of Table 8a shows that the F1 score of the MPGL model improved by 1.2% compared with the baseline SPSL. In the evaluation of Table 8b, when the SER model was trained on KESDy18_EM and IMEOCAP datasets, which were from different language cultures, the model was evaluated using the Korean KESDy18_PM domain dataset. The proposed MPGLN SER showed an F1-score improvement of about 3.5% over the baseline model.

**Figure 5:**
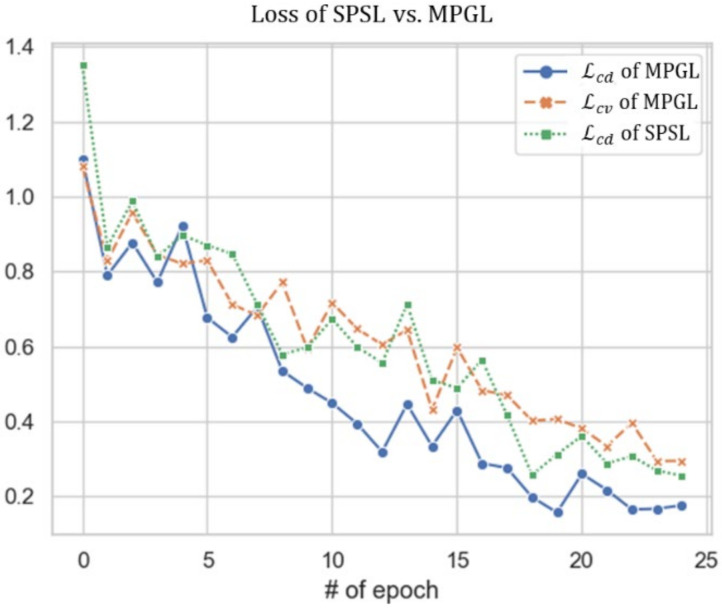
Change in losses of the baseline SER and the proposed MPGLN SER in Table 8b. The loss, $L_{cd}$, of the baseline SPSL model and losses $L_{cd}$ and $L_{cv}$ of the SER model of MPGL.

Figure 5 shows the changes in losses from Table 8b, including the loss, $L_{cd}$, of the baseline SPSL model and losses $L_{cd}$ and $L_{cv}$ of the MPGL SER model. These losses were measured every 25 epochs during training using aggregated KESDy18_EM and IEMOCAP samples. The loss, $L_{cd}$, of the MPGL model, which learned two losses simultaneously, trained faster than did the $L_{cd}$ of the baseline SER model. This shows that the other complementary loss, $L_{cv}$, of the proposed MPGLN, used to predict the valence-level label, decreased similarly to the loss, $L_{cd}$, of the baseline SPSL.

**Figure 6:**
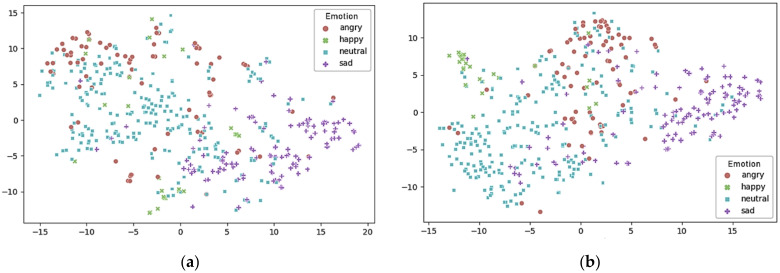
Distribution of reduced embedding vectors (the 64-D embedding vectors of the test data in the last fully-connected (FC) layer in the ensemble network) that are reduced to 2-D via *t*-stochastic neighbor embedding (t-SEN) dimension reduction: (**a**) embedding space for MPSL in Table 8b; (**b**) embedding space for MPGL in Table 8b.

Figure 6 shows the distribution of the 64-D embedding vectors of the test data reduced to a 2-D embedding space via *t*-stochastic neighbor embedding (t-SEN). The 64-D embedding vectors were generated in the FC layer just prior to the MPSL and MPGL softmax activations of the evaluation in Table 8b. 

Figure 6a shows the distribution of the embedding feature vector in the MPSL trained by the loss, $L_{cd}$, only without the complementary loss, $L_{cv}$. Figure 6b displays the distribution of the MPGL model based on the loss group, $L_{g}$, of the two losses: $L_{cd}$ and $L_{cv}$. Figure 6b shows the MPGLN SER model that learns from multi-path embedding vectors and the loss group, $L_{g}$, where the samples belonging to the “happy” class were more closely grouped, and the samples of the “angry” and “sad” classes are located closer together compared with the MPSL distribution shown in Figure 6a.

## 5. Conclusions

We determined that it is essential to improve the generalization of the SER model for deployment to real applications. This paper proposed the MPGLN for SER in support of supervised multi-domain adaptation and generalization based on multi-domain datasets. The proposed MPGLN SER includes a temporal feature generator for the BLSTM network using the input of handcrafted LLD features of a speech sample. Additionally, we leveraged the transferred feature extractor from the pre-trained VGGish model for the MPGLN. The proposed MPGLN SER learned simultaneous multiple losses induced by associations between discrete emotion and dimension labels.

The proposed MPGLN SER was evaluated using five real SER datasets of various speaker domains, language cultures, collecting devices, and procedural environments. This included KESDy18 and KESDy19 databases. KESDy18 comprised speech samples delivered by voice actors who uttered Korean short sentences by expressing specific discrete emotions. The KESDy18 database consisted of KESDy18_PM and KESDy18_EM datasets from heterogeneous devices and environments with different device locations. The KESDy19 database comprised KESDy19_EM and KESDy19_PM, which contained the collected speech sample voices acted using a similar procedure as that of the IEMOCAP and that of the simulated dataset based on the cell-phone’s built-in microphone, respectively.

This study assumed that the SER model was trained only with the BLSTM-based temporal embedding feature generator included with MPGLN without transferred feature as the baseline SER model. We verified the performance reliability of the baseline SER model using the IEMOCAP. The BLSTM-baseline SER model showed competitive UA results of 59% when classifying the four categorical emotion labels. The multi-domain adaptation and domain generalization evaluation of the proposed MPGLN SER was performed using the English-speaking IEMOCAP and the Korean KESDy18 and KESDy19 datasets by comparing the performances of the baseline model according to various evaluation environments.

The proposed MPGLN SER model trained on multiple losses showed an F1 performance improvement of up to 3.7% over the baseline model when classifying four emotion labels in a single domain dataset. The performance evaluation of the MPGLN SER for supervised multi-domain adaptation, which trained and tested on the SER model using the aggregated speech samples of the multi-domain datasets, also showed an improvement of up to 3.7% over the baseline F1 score. From the evaluation of the multi-domain generalization of the proposed MPGLN SER, the F1 score enjoyed an improvement of 3.5% over the baseline SER when using samples from other language cultures not used for training. From these results, we found that our MPGLN SER, which supports supervised multi-domain adaptations, is also effective in reinforcing the generalization of the SER model based on multi-domain datasets.

For future works, we plan to derive the differences in acoustic features of emotional expressions based on multi-cultural SER datasets and study the learning method for the deep-learning-based SER model considering the domain discrepancy. Furthermore, we will continue enhancing our model’s generalizability through evaluations of speech data in the wild by deploying the proposed MPGLN SER to real applications.

## Figures and Tables

**Table 1:** Association properties of discrete emotion labels and valence-levels in multi-domain SER datasets: (**a**) Mean and standard variation of arousal and valence levels on a five-point scale for each discrete emotion category; (**b**) Confidence of discrete emotion labels and valence-level of three-point scale.

Index	Association Property		IEMOCAP	KESDy18	KESDy19
(a)	Valence Mean ± variation	angry	1.89 ± 0.52	2.11 ± 0.21	1.78 ± 0.37
		happy	3.94 ± 0.47	4.42 ± 0.34	4.33 ± 0.36
		neutral	2.95 ± 0.49	3.23 ± 0.53	2.94 ± 0.60
		sad	2.24 ± 0.57	2.00 ± 0.33	1.89 ± 0.52
	Arousal Mean ± variation	angry	3.69 ± 0.66	3.93 ± 0.46	3.81 ± 0.58
		happy	3.16 ± 0.61	3.92 ± 0.36	3.90 ± 0.53
		neutral	2.79 ± 0.53	3.08 ± 0.38	2.99 ± 0.33
		sad	2.61 ± 0.61	2.60 ± 0.44	2.63 ± 0.64
(b)	Confidence	*Conf*.($\left\{C_{i}=angry\right\}$->{$V_{1}$})	0.8	0.95	0.95
		*Conf*.($\left\{C_{i}=sad\right\}$->{$V_{1}$})	0.58	0.9	0.86
		*Conf*.($\{C_{i}=neutral$}->{$V_{2}$})	0.85	0.83	0.71
		*Conf*.($\left\{C_{i}=happy\right\}$->{$V_{3}$})	0.77	0.93	0.86

**Table 2:** Properties of multi-domain SER datasets.

Property	IEMOCAP	KESDy18	KESDy19 ^2^
Language	English	Korean	Korean
Speakers	10 (5 male, 5 female)	30 (15 male, 15 female)	40 (20 male, 20 female)
Utterance type	Acted (Scripted/Improvised)	Acted (Scripted)	Acted (Scripted/Improvised)
Datasets (Mic.)	IEMOCAP (2 Mic. of the same type)	KESDy18_PM (Galaxy S6), KESDy18_EM ^1^ (Shure S35)	KESDy19_PM (Galaxy S8), KESDy19_EM (AKG C414)
angry	947	431	1628
happy	507	157	1121
neutral	1320	1193	2859
sad	966	467	694
Total	3740	2248	6302

^1^ KESDy18_EM is available online at https://nanum.etri.re.kr/share/kjnoh/SER-DB-ETRIv18?lang=eng (accessed on 7 January 2021). ^2^ The collecting process of the KESDy19 was approved by the Institutional Review Board of Korea National Institute for Bioethics Policy (approval number P01-201907-22-010 and 22 July 2019).

**Table 3:** Performance of the baseline BLSTM-based SER model according to the input low-level descriptions (LLD) feature set in SER datasets.

Model	Dataset	Input LLDs	WA	UA	PR	F1
Our baseline (SPSL: single-path-single-loss)	IEMOCAP	MFCC	0.616	0.588	0.576	0.559
		Mel-spec	0.534	0.525	0.504	0.491
		MFCC + Mel-spec	0.608	0.58	0.574	0.562
		MFCC + Mel-spec + TimeSpectral	0.611	0.59	0.58	**0.575**
	KESDy18_EM	MFCC	0.742	0.712	0.715	0.71
		Mel-spec	0.62	0.57	0.553	0.556
		MFCC + Mel-spec	0.762	0.736	0.719	0.724
		MFCC + Mel-spec + TimeSpectral	0.774	0.738	0.737	**0.734**
	KESDy19_EM	MFCC	0.613	0.563	0.581	0.567
		Mel-spec	0.56	0.483	0.518	0.491
		MFCC + Mel-spec	0.617	0.562	0.579	0.568
		MFCC + Mel-spec + TimeSpectral	0.643	0.595	0.608	**0.599**

**Table 4:** Performance of the baseline BLSTM-based SER model.

Model	Dataset	WA	UA	PR	F1
Our baseline (SPSL)	IEMOCAP	0.611	0.59	0.58	0.575
	KESDy18_PM	0.776	0.739	0.739	0.736
	KESDy18_EM	0.774	0.738	0.737	0.734
	KESDy19_PM	0.624	0.574	0.589	0.58
	KESDy19_EM	0.643	0.595	0.608	0.599

**Table 5:** Performance results reported in previous recurrent neural networks (RNN)-based studies of SER model and our baseline model based on IEMOCAP.

Researches	Features	Network	UA	Emotions
Mirsamadi [19]	32 LLD	RNN	0.585	4
Chen ^1^ [20]	logMel	CRNN	0.647 ± 0.054	4
Mu [26]	Spectrogram	CRNN	0.564	4
Our baseline (SPSL)	74 LLD	RNN	0.59 ± 0.08	4

^1^ This study used only the improvisation data of female speakers as test data.

**Table 6:** Evaluation results in a single domain dataset. Single-path-single-loss (SPSL) is the baseline SER model that learns by the temporal embedding features and the loss $L_{cd}$; Multi-path-single-loss (MPSL) is that model learns using the multi-path embedding vectors and loss $L_{cd}$ without the loss $L_{cv}$; MPGL is the model that learns based on multi-path embedding vectors and the group loss $L_{g}$.

Index	Domain	Model	WA	UA	PR	F1
(a)	IEMOCAP	SPSL	0.611	0.59	0.58	0.575
		MPSL	0.611	0.606	0.576	0.583
		MPGL	0.619	0.607	0.582	**0.588**
(b)	KESDy18_PM	SPSL	0.776	0.739	0.739	0.736
		MPSL	0.781	0.753	0.747	0.746
		MPGL	0.814	0.778	0.771	**0.773**
(c)	KESDy18_EM	SPSL	0.774	0.738	0.737	0.734
		MPSL	0.788	0.756	0.732	0.741
		MPGL	0.797	0.768	0.761	**0.762**
(d)	KESDy19_PM	SPSL	0.624	0.574	0.589	0.58
		MPSL	0.625	0.581	0.594	0.586
		MPGL	0.637	0.586	0.607	**0.594**
(e)	KESDy19_EM	SPSL	0.643	0.595	0.608	**0.599**
		MPSL	0.629	0.581	0.591	0.584
		MPGL	0.642	0.592	0.608	0.598

**Table 7:** Evaluation results of multi-domain adaptation.

Index	Multi-Domain	Model	WA	UA	PR	F1
(a)	KESDy18_PM, KESDy18_EM	SPSL	0.774	0.749	0.722	0.731
		MPSL	0.799	0.764	0.753	0.756
		MPGL	0.806	0.773	0.766	**0.768**
(b)	KESDy19_PM, KESDy19_EM	SPSL	0.618	0.581	0.584	0.581
		MPSL	0.626	0.58	0.589	0.584
		MPGL	0.631	0.585	0.595	**0.589**
(c)	KESDy18_PM, KESDy18_EM, KESDy19_PM, KESDy19_EM	SPSL	0.653	0.628	0.63	0.628
		MPSL	0.664	0.639	0.642	**0.639**
		MPGL	0.663	0.63	0.639	0.634
(d)	KESDy18_PM, KESDy18_EM, IEMOCAP	SPSL	0.683	0.649	0.63	0.637
		MPSL	0.706	0.675	0.654	0.66
		MPGL	0.713	0.677	0.656	**0.664**
(e)	KESDy19_PM, KESDy19_EM, IEMOCAP	SPSL	0.599	0.577	0.575	0.573
		MPSL	0.602	0.583	0.576	0.578
		MPGL	0.616	0.587	0.59	**0.588**

**Table 8:** Evaluation results of multi-domain generalization.

Index	Source Domain	Target Domain	Model	WA	UA	PR	F1
(a)	KESDy18_PM, KESDy18_EM, KESDy19_EM	KESDy19_PM	SPSL	0.594	0.532	0.563	0.539
			MPSL	0.592	0.53	0.559	0.536
			MPGL	0.606	0.543	0.573	**0.551**
(b)	KESDy18_EM, IEMOCAP	KESDy18_PM	SPSL	0.682	0.69	0.652	0.658
			MPSL	0.688	0.704	0.643	0.658
			MPGL	0.718	0.74	0.677	**0.693**
(c)	KESDy19_EM, IEMOCAP	KESDy19_PM	SPSL	0.572	0.55	0.538	0.538
			MPSL	0.577	0.552	0.545	0.542
			MPGL	0.596	0.555	0.561	**0.554**

## Data Availability

Statistical results are contained within the article. The KESDy18_EM dataset collected in this study is available online at https://nanum.etri.re.kr/share/kjnoh/SER-DB-ETRIv18?lang=eng (accessed on 7 January 2021).
